# Cultivation of common bacterial species and strains from human skin, oral, and gut microbiota

**DOI:** 10.1186/s12866-021-02314-y

**Published:** 2021-10-14

**Authors:** Elizabeth Fleming, Victor Pabst, Zoe Scholar, Ruoyun Xiong, Anita Y. Voigt, Wei Zhou, Amelia Hoyt, Rachel Hardy, Anna Peterson, Ryan Beach, Yvette Ondouah-Nzutchi, Jinhong Dong, Lucinda Bateman, Suzanne D. Vernon, Julia Oh

**Affiliations:** 1 The Jackson Laboratory, 10 Discovery Drive, Farmington, CT 860-837-2014 USA; 2 The University of Connecticut Health Center, Farmington, CT USA; 3 Bateman Horne Center, Salt Lake City, UT 84102 USA

## Abstract

**Background:**

Genomics-driven discoveries of microbial species have provided extraordinary insights into the biodiversity of human microbiota. In addition, a significant portion of genetic variation between microbiota exists at the subspecies, or strain, level. High-resolution genomics to investigate species- and strain-level diversity and mechanistic studies, however, rely on the availability of individual microbes from a complex microbial consortia. High-throughput approaches are needed to acquire and identify the significant species- and strain-level diversity present in the oral, skin, and gut microbiome. Here, we describe and validate a streamlined workflow for cultivating dominant bacterial species and strains from the skin, oral, and gut microbiota, informed by metagenomic sequencing, mass spectrometry, and strain profiling.

**Results:**

Of total genera discovered by either metagenomic sequencing or culturomics, our cultivation pipeline recovered between 18.1–44.4% of total genera identified. These represented a high proportion of the community composition reconstructed with metagenomic sequencing, ranging from 66.2–95.8% of the relative abundance of the overall community. Fourier-Transform Infrared spectroscopy (FT-IR) was effective in differentiating genetically distinct strains compared with whole-genome sequencing, but was less effective as a proxy for genetic distance.

**Conclusions:**

Use of a streamlined set of conditions selected for cultivation of skin, oral, and gut microbiota facilitates recovery of dominant microbes and their strain variants from a relatively large sample set. FT-IR spectroscopy allows rapid differentiation of strain variants, but these differences are limited in recapitulating genetic distance. Our data highlights the strength of our cultivation and characterization pipeline, which is in throughput, comparisons with high-resolution genomic data, and rapid identification of strain variation.

**Supplementary Information:**

The online version contains supplementary material available at 10.1186/s12866-021-02314-y.

## Background

Genomics-driven innovations, such as high-throughput 16S ribosomal RNA (rRNA) gene and whole-genome shotgun metagenomic sequencing have been powerful drivers of discovery in a wide range of microbial ecosystems, including those that impact human health. The blueprints created by large-scale studies have elucidated an extraordinary microbial biodiversity across individuals, geographies, ethnicities, disease states, and lifestyles [1]. Such blueprints have been critical for baseline characterizations of different ecosystems and generating hypotheses by correlative analyses with phenotypes of interest [2, 3]. The logical next step for understanding host microbiome interactions are mechanistic investigations that are potentiated by findings from metagenomic surveys, but made possible by possessing the substrates of interest – namely, the microbes themselves.

Obtaining microbial isolates from a sample of interest has multiple values. First, sequencing isolates provides the highest quality reference genome sequences; genome reconstructions from metagenomic data can result in incomplete and fragmented genomes [4–6], as closely related genomes, low abundance genomes, and highly complex communities pose significant computational challenges that hinder accurate reconstruction of function and biodiversity [7]. Second, an isolate in hand allows experimentation and manipulation to understand its genetics, molecular and physiological mechanisms, and inter- and intra-species interactions. Third, isolates allow precise investigation of strain diversity, which is critical as individual strains of a microbial species can exhibit widely diverse phenotypes [7]. For example, most *Escherichia (E.) coli* strains are commensals in the human gastrointestinal tract, while some strains can cause severe disease [8]. In addition, isolates are necessary for inferring evolutionary dynamics, transmission, and lineage tracking during infectious disease outbreaks [9]. Finally, a tremendous genetic and phenotypic diversity is encoded at the strain level, rather than higher taxonomic levels, and individual disease susceptibility or severity phenotypes can be attributed to not only a common species, but unique strains [8, 10]. Different algorithms have been developed to infer strain diversity from metagenomic datasets, e.g. [11–13], but these can vastly underestimate strain diversity. Thus, cultivation of microbiota accounting for strain diversity would facilitate investigation of patient-specific genotypes and phenotypes.

Finally, systematic methods for cultivation and recovery of microbial isolates from a sample is complicated by the notable site specificity of the human microbiome [14–16]. For example, the gut harbors the highest biodiversity, with characteristic bacteria from Bacteroides and varied lactobacilli, enterobacilli and enterococci, *Bifidobacteria*, Clostridia, and methanogens [14]. The oral cavity is typically populated with streptococci, *Haemophilus*, *Prevotella*, *Veillonella* genera, and the skin staphylococci with *Corynebacterium* and *Cutibacterium* [14]. Even within each of these body sites, significant local variation exists, such as the stomach vs. the small intestine vs. the cecum [17], oral pockets vs. the dorsum of the tongue [18, 19], or the moist, oily, dry, or foot sites of the skin [20].

Numerous approaches have been defined to systematically cultivate microbes from different ecosystems, with a focus particularly on the gut [21, 22]. Extraordinary efforts have been made to increase the recovery of gut microbial biodiversity from the anaerobic environment of the gut, using up to 212 different culture conditions [21, 23], which might include a wide variety of different nutritive conditions or additives, different gas fractions, temperatures, pH, or inhibition via antimicrobials. Microfluidics devices [24] for optimized isolation of cells, or metagenomic prediction of membrane epitopes for synthetic design of antibodies have also been used to capture microorganisms of interest [25]. Multiple, sequenced large-scale gut microbial culture collections have been recently established [21, 26–28], and these efforts have correspondingly increased the accurate annotation of metagenomic datasets. In contrast, while skin cultivation methods were prolific in the 1950s [29], there was no potential to inform recovery using metagenomic characterizations, and fewer consolidated and systematic efforts exist for human oral or skin microbial cultivation, with recent efforts primarily targeted efforts to recover microbes of interest, like *Cutibacterium acnes* [30] or Gram negatives [31].

Here, our ultimate goals were to define a set of user-friendly cultivation conditions that would allow us to 1) culture dominant microbiota from many different individuals and body sites, 2) estimate recovery based on metagenomic data, and importantly, 3) identify rapid, low-cost approaches to delineate strain diversity, which would facilitate isolate choice for more laborious and costly whole genome sequencing. We cultivated isolates from the human gut, oral cavity, and two physiologically diverse skin sites on a streamlined set of different nutritive conditions. We performed shotgun sequencing of the same sample to evaluate the proportion of microbes recovered by cultivation. Finally, we compared the ability of Fourier-Transform Infrared Spectroscopy (FT-IR) to rapidly classify and differentiate strains of common species with whole genome sequencing. Taken together, this work builds on and consolidates approaches for generating culture collections from a variety of different environments, enabling a range of follow-up genomic and phenotypic characterizations.

## Results

### Sample description

Our goal was to identify a core set of cultivation conditions for each body site that would 1) allow recovery of dominant microbes from a large number of samples, which 2) would recover strain variants of these species. In addition, we sought to evaluate how well these conditions promoted culture of the microbial diversity of the sample, as we anticipated that approaches favoring throughput would limit a comprehensive recovery of microbes from each body site. For our sample choice, we obtained human samples from the following sites: for the skin, we chose the forehead and toe web space as representatives of an oily and a moist skin site, respectively, and their microbiota differ markedly in our previous surveys [7, 15, 20]. We chose the inner cheek and tongue dorsum to represent the oral cavity, and stool for gut. Because different individuals can harbor markedly different microbial species and strains [14], we obtained a total of 25 samples (5 samples per body site) from 12 individuals. For each sample, we then performed metagenomic shotgun sequencing (1.6 ± 1.0 × 10^6^, 2.0 ± 1.1 × 10^6^, 10.5 ± 1.0 × 10^6^ quality-controlled, human DNA dehosted reads for oral, skin, and stool, respectively, Table S1) and culturomics as described below.

### Cultivation conditions and species identification

**Fig. 1:**
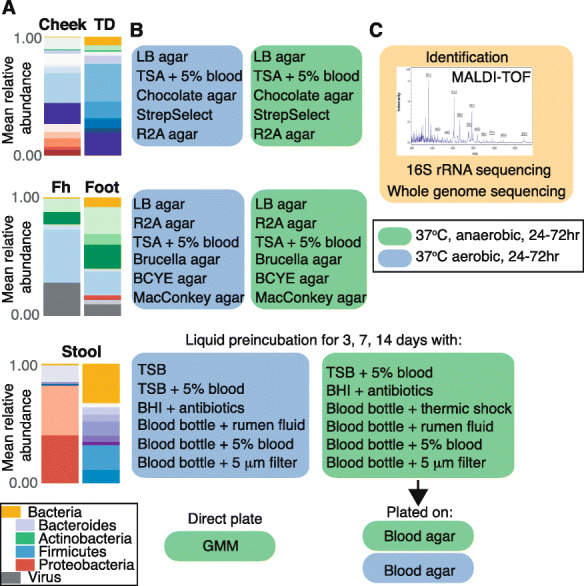
Culturomics pipeline. **A)** Metagenomic data were generated for each oral (TD: tongue dorsum), skin (Fh: forehead), or stool sample. Example (non-representative) relative abundance plots of major species are shown with colors corresponding to phylum as shown in the legend. Samples were then **B)** diluted and cultivated in a defined set of anaerobic (green boxes) and aerobic (blue boxes) cultivation conditions for oral, skin, and stool. After a defined period of incubation, individual colonies were picked, subcultured for purity, then **C)** identified using MALDI-TOF, 16S rRNA gene sequencing, or whole genome sequencing

**Fig. 2:**
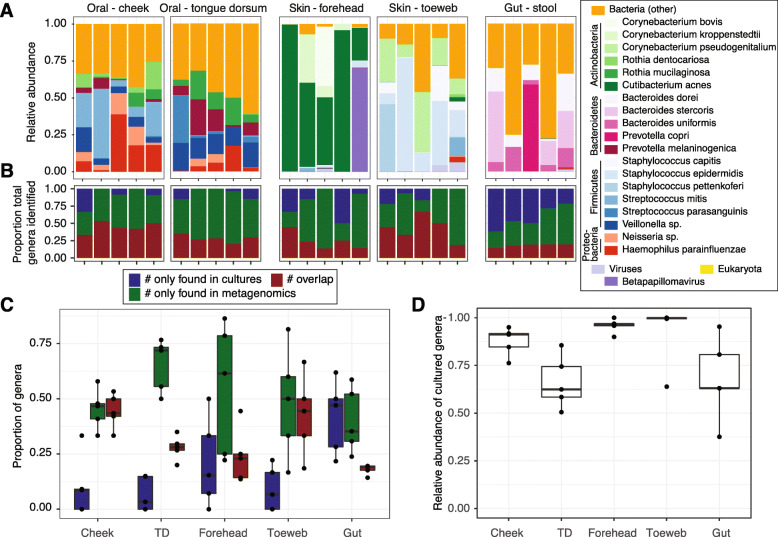
Metagenomic reconstructions of community composition and representation by cultivars. **A)** Relative abundance plots of oral, skin and gut samples from this study; each bar is an individual sample and the top 20 most abundant species are plotted. Lesser abundance bacteria, fungi, and viruses are collectively represented by their respective kingdom. Figure S1 visualize higher taxonomic levels. The proportion of bacterial genera cultivated or identified through sequencing for each sample, **B),** or across samples shown by boxplot, **C)**. Red shows the proportion of bacterial genera identified through both cultivation and metagenomics, and blue and green show the proportion of bacterial genera identified by only one method. **D)** The total relative abundance of the original metagenomic sample (bacteria only) that is accounted for by the genera cultivated (overlap in **C**)

Guided by our previous metagenomic data and a literature search [21, 22, 26, 27, 29–46], we compiled the aerobic and anaerobic cultivation conditions reported in Table 1 and Fig. 1. To examine the proportion of microbes recovered by these conditions, we first characterized the fungal, bacterial, and viral composition of our samples using shotgun metagenomics as it is culture-independent and yields the most unbiased compositional reconstruction (Fig. 2, Fig. S1, Table S2). Consistent with previous reports [14, 15], *Cutibacterium acnes* and *Corynebacterium* sp. were most abundant in the oily sites of the forehead, and staphylococci and *Corynebacterium* sp. in the foot. In the cheek, streptococci, *Rothia mucilanginosa*, and *Haemophilus parainfluenzae* were most abundant, and in the tongue dorsum, *Neisseria flavescens, Prevotella* sp., *Veillonella* sp.*,* and streptococci. Finally, in the stool samples, *Bacteroidales* and *Clostridiales* were most abundant.

**Table 1:** Cultivation conditions

Skin cultivation conditions	
Media	Conditions
LB agar	Aerobic, Anaerobic, 37 °C
R2A agar	Aerobic, Anaerobic, 37 °C
TSA with 5% sheep blood	Aerobic, Anaerobic, 37 °C
Brucella agar	Aerobic, Anaerobic, 37 °C
BCYE agar	Aerobic, Anaerobic, 37 °C
MacConkey agar	Aerobic, Anaerobic, 37 °C
**Oral cultivation conditions**	
**Media**	**Conditions**
LB agar	Aerobic, Anaerobic, 37 °C
R2A agar	Aerobic, Anaerobic, 37 °C
TSA with 5% sheep blood	Aerobic, Anaerobic, 37 °C
Chocolate agar	Aerobic, Anaerobic, 37 °C
Selective Strep agar	Aerobic, Anaerobic, 37 °C
**Stool cultivation conditions**	
**Culture device prior to plating onto TSA with 5% sheep blood**	**Conditions**
Direct plating onto GMM agar	Anaerobic, 37 °C
TSB (3, 7, 14d) + sheep blood (9% final vol)	Aerobic, 28 °C
Aerobic blood bottle (3, 7, 14d) + rumen fluid (9% final vol)	Aerobic, 37 °C
Aerobic blood bottle (3, 7, 14d) + sheep blood (9% final vol)	Aerobic, 37 °C
Aerobic blood bottle (3, 7, 14d); sample filtered at 5 μm	Aerobic, 37 °C
BHI (3, 7, 14d) + vancomycin + colistin (10 μg/mL each)	Aerobic, 37 °C
TSB (3, 7, 14d)	Aerobic, 37 °C
Anaerobic Blood bottle (3, 7, 14d) + sheep blood (9% final vol)	Anaerobic, 37 °C
Anaerobic Blood bottle (3, 7, 14d) + rumen fluid (9% final vol)	Anaerobic, 37 °C
Anaerobic Blood bottle (3, 7, 14d); sample filtered at 5 μm	Anaerobic, 37 °C
Anaerobic Blood bottle (3, 7, 14d); thermic shock (85 °C, 20 min)	Anaerobic, 37 °C
BHI (3, 7, 14d) + vancomycin + colistin (10 μg/mL each)	Anaerobic, 37 °C
TSB (3, 7, 14d) + sheep blood (9% final vol)	Anaerobic, 37 °C

In culturing, we recommend a rule of thumb to dilute samples 1:10 and 1:100 for skin sites, 1:100 and 1:1000 for oral sites, 1:1000 and 1:10000 for gut samples prior to plating, reflecting the low, medium, and high microbial bioburden of these sites, respectively. Dilution plates usually consisted of 1–4 dominant microbes with singletons interspersed at low density. Because of this we aimed to select ~ 12 microbes per plate, selecting up to 3 of each visibly unique morphology for subculturing to further purify the selected isolate, followed by MALDI-TOF mass spectrometry analysis for identification. MALDI-TOF accuracy at the species and genus level varies widely by taxonomy and even instrument (e.g., ~ 84% for species, ~ 92% for genus in a recent estimate of anaerobic bacteria [47], but up to 98% accuracy and 94% accuracy can be observed in *Enterobacteriaceae* and staphyloccci [48]), with disease-associated species having the deepest reference databases and thus the highest corresponding accuracy. Thus, we emphasize here the comparisons at the genus level, but also report species-level results, recognizing that discrimination at the species level is critical for many human-associated pathobionts, such as staphylococci, which encompass disease-causing *S. aureus* vs. many commensal species. Overall, we obtained 15 unique genera (34 species) in the skin, 17 genera (53 species) in the mouth, and 41 genera (97 species) from stool from 600, 1155, and 1451 isolates tested, respectively (Table 2, Table S3, species-level in Table S4). Some bacteria were ubiquitous (isolated from nearly every condition, e.g., *Staphylococcus* in skin, *Streptococcus* in oral, *Enterococcus and Escherichia* in stool, Table S3**,** species in Table S4). Other microbes had more restricted recovery; for example, *Bifidobacterium* sp. were only isolated at early timepoints (< 3 days, Table S3, Table S4), while recovery of *Anaerococcus* sp. was achieved only at later timepoints (>14d), though we note that these conclusions are impacted by colony sampling and is meant to provide rules of thumb.

**Table 2:** Genera uniquely detected by culturomics, metagenomics, and overlap

Phylum	Genus	# samples cultured only	# samples metagenomic only	# overlaps	# total samples
Actinobacteria	Corynebacterium	5	2	12	19
Firmicutes	Streptococcus	1	7	11	19
Firmicutes	Veillonella	0	7	10	17
Proteobacteria	Neisseria	0	4	10	14
Actinobacteria	Actinomyces	2	3	10	15
Actinobacteria	Rothia	0	6	9	15
Firmicutes	Staphylococcus	8	0	9	17
Actinobacteria	Cutibacterium	1	3	8	12
Proteobacteria	Haemophilus	0	9	7	16
Firmicutes	Gemella	0	7	6	13
Firmicutes	Lachnoanaerobaculum	0	5	5	10
Bacteroidetes	Bacteroides	0	1	5	6
Bacteroidetes	Parabacteroides	0	0	5	5
Fusobacteria	Fusobacterium	1	3	4	8
Firmicutes	Lactobacillus	2	2	3	7
Actinobacteria	Bifidobacterium	0	1	3	4
Actinobacteria	Collinsella	0	0	3	3
Bacteroidetes	Prevotella	0	12	2	14
Proteobacteria	Aggregatibacter	0	6	2	8
Firmicutes	Clostridium	3	4	2	9
Actinobacteria	Micrococcus	2	2	2	6
Proteobacteria	Escherichia	3	1	2	6
Actinobacteria	Brevibacterium	1	1	2	4
Firmicutes	Flavonifractor	2	0	2	4
Firmicutes	Granulicatella	0	13	1	14
Actinobacteria	Atopobium	1	5	1	7
Actinobacteria	Kocuria	1	5	1	7
Firmicutes	Eubacterium	1	3	1	5
Proteobacteria	Acinetobacter	0	3	1	4
Bacteroidetes	Alistipes	0	3	1	4
Firmicutes	Blautia	1	2	1	4
Bacteroidetes	Odoribacter	1	2	1	4
Actinobacteria	Kytococcus	1	1	1	3
Firmicutes	Bacillus	5	0	1	6
Actinobacteria	Dermabacter	2	0	1	3
Bacteroidetes	Capnocytophaga	0	9	0	9
Proteobacteria	Actinobacillus	0	8	0	8
Fusobacteria	Leptotrichia	0	7	0	7
Bacteroidetes	Porphyromonas	0	7	0	7
Bacteroidetes	Alloprevotella	0	7	0	7
Firmicutes	Oribacterium	0	6	0	6
Firmicutes	Stomatobaculum	0	6	0	6
Firmicutes	Subdoligranulum	0	6	0	6
Proteobacteria	Campylobacter	2	5	0	7
Firmicutes	Peptostreptococcus	2	5	0	7
Firmicutes	Megasphaera	0	5	0	5
Firmicutes	Solobacterium	0	5	0	5
Firmicutes	Oscillibacter	0	5	0	5
Proteobacteria	Enhydrobacter	0	4	0	4
Firmicutes	Abiotrophia	0	4	0	4
Proteobacteria	Kingella	0	4	0	4
Firmicutes	Dorea	0	4	0	4
Proteobacteria	Bilophila	0	4	0	4
Firmicutes	Faecalibacterium	0	4	0	4
Firmicutes	Finegoldia	2	3	0	5
Firmicutes	Selenomonas	0	3	0	3
Saccharibacteria	Saccharibacteria	0	3	0	3
Proteobacteria	Parasutterella	0	3	0	3
Firmicutes	Roseburia	0	3	0	3
Firmicutes	Anaerococcus	3	2	0	5
Firmicutes	Ruminococcus	2	2	0	4
Firmicutes	Parvimonas	1	2	0	3
Actinobacteria	Eggerthella	3	1	0	4
Firmicutes	Pediococcus	2	1	0	3
Firmicutes	Enterococcus	6	0	0	6
Proteobacteria	Citrobacter	3	0	0	3
Actinobacteria	Dietzia	3	0	0	3
Firmicutes	Lysinibacillus	3	0	0	3

We then sought to examine the degree to which these isolates represented the predicted composition from metagenomic data. At the genus level (to account for MALDI-TOF and metagenomic classification accuracy at the species level), we observed an overlap of genera cultivated vs. sequenced of 44.4 ± 7.7% (cheek, mean ± standard deviation), 27.9 ± 5.4% (tongue dorsum), 24.1 ± 12.4% (forehead), 24.1 ± 18.0% (toeweb), and 18.1 ± 2.3% (stool, Fig. 2B-C). These represented a high proportion of the community composition reconstructed with metagenomic sequencing (Fig. 2D); 87.7 ± 7.5%, 66.2 ± 13.8%, 95.8 ± 3.7%, 92.6 ± 16.1%, and 67.9 ± 21.7%, respectively, suggesting that our methods captured the majority of the abundant genera irrespective of body site and that many of the missing genera were potentially low abundance microbes. Correlation analysis between relative abundance and frequency of cultivation (number of times an isolate was identified), by body site, showed a range of associations (species- and genus-level Spearman’s rho and *p*-values reported in Table S5, example scatterplots in Fig. S2). Some species showed a positive correlation (e.g., *Staphylococcus* sp. *lugdunensis, pettenkoferi, warneri*, although at the genus level *Staphylococcus* was not positively associated), and in some cases a significant negative association (e.g., *E. coli*), reflecting low relative abundance but high frequency of cultivation.

We then examined if, and what genera were preferentially cultivated and found there were genera that were identified only by metagenomic sequencing but also only by cultivation (Table 2, Fig. 2B-C, Table S5 for species and genus-level). Metagenomics, as expected, definitively recovered a larger number of total microbes that were not captured by culturomics (Fig. 2B). Prevalent metagenomic genera that were not cultured were primarily anaerobes from the gut and oral cavity, including *Capnocytophaga* (phylum Bacteroidetes, found in 9 samples), *Porphyromonas* [7], *Alloprevotella* [7]*, Actinobacillus* (phylum Proteobacteria, 7), and *Leptotrichia* (phylum Fusobacteria, 7). We were also surprised that we consistently identified species that were not captured by metagenomics. Interestingly, *Enterococcus* (phylum Firmicutes) was never identified by sequencing, but fairly extensively cultured as several species (*E. avium, faecalis, faecium* across six samples)*.* Similarly, *Bacillus* species (*B. circulans, pumilus, subtilis*) were only identified in one metagenomic sample (as *Bacillus amyloliquefaciens*) compared to five samples for culturomics. Even well-studied bacteria like staphylococci could be cultivated from a sample more frequently than detected by metagenomic data; 8/17 times it was only detected by cultivation. Potential explanations for these observations could include: 1) A species is easily cultivatable (e.g., staphylococci, *Enterococcus*) but low abundance, resulting in insufficient reads that can be classified to a species by algorithms such as Metaphlan2 [49], which map reads to a limited set of species-specific marker genes, 2) incomplete or few reference genomes are available for that genus to enable its classification (e.g., *Bacillus* sp.*,* which have relatively fewer reference genomes compared to *Enterococcus* with hundreds of deposited reference genomes). This would also result in a limited ability of Metaphlan2-like algorithms that identify clade-specific marker genes to identify robust discriminatory features. Here, mapping of metagenomic data selectively to *Enterococcus* reference genomes confirmed a very low mapping rate and thus relative abundance in the sample.

### Strain identification

An emerging frontier of metagenomic discovery is the understanding of strain biology, as microbial diversity is ultimately manifested at this finest taxonomic resolution where individual strains of a microbial species can exhibit widely diverse phenotypes. As our methods primarily recover dominant microbes (e.g., staphylococci, *E. coli*), it is particularly effective for investigating strain variation between individuals and cohorts. Further, extensive strain variation can exist not only between, but as we have shown, within individuals [7]. This phenomenon has major implications for disease severity [7]. It is thus valuable to be able to rapidly differentiate strains to understand strain diversity, to identify disease-causing strains, and to prioritize strains for phenotyping.

Different methods with widely differing resolution have been developed for strain typing primarily for clinical use, perhaps most commonly multi-locus sequence typing (MLST) [50], which sequences polymorphisms in highly conserved genes to bin strains into sequence ‘types’. The gold standard is whole genome sequencing, but despite extraordinary technical advances, it remains relatively costly and slow to perform and analyze on large scales. Other nucleic acid-based approaches, such as Rep-PCR, leverage strain-specific differences in repetitive regions to discriminate (but not identify) strains, but can be time consuming to deploy with multiple rounds of PCR, electrophoresis, and interpretation [51, 52]. FT-IR’s promise is in its low-cost and rapid generation of discriminatory biochemical fingerprints primarily based on cell surface macromolecules, e.g., lipids, proteins, and carbohydrates. It has been used in examining clonal outbreaks of varying origin, although overall it remains less frequently used despite its lengthy technical history (reviewed in [53]), likely because of incomplete understanding of the link between genetic diversity and cell surface macromolecular diversity.

Here, we evaluated the ability of the Bruker IR Biotyper to rapidly differentiate genetically diverse strains from phylogenetically diverse species, selected as common species of interest in the skin, oral, or gut microbiota. In addition to cultivars obtained in our study, we included additional publicly available, fully sequenced isolates to provide additional genetic diversity. Finally, by way of benchmark, we sequenced, or obtained from public repositories, the genomes of these strains to determine genetic relatedness (Table S1), although we recognize that the cell surface macromolecules are encoded and modified by numerous genetic pathways and environmental conditions, like length of growth time and composition of growth media, and are likely difficult to translate to genetic distance, as previously noted [53].

**Fig. 3:**
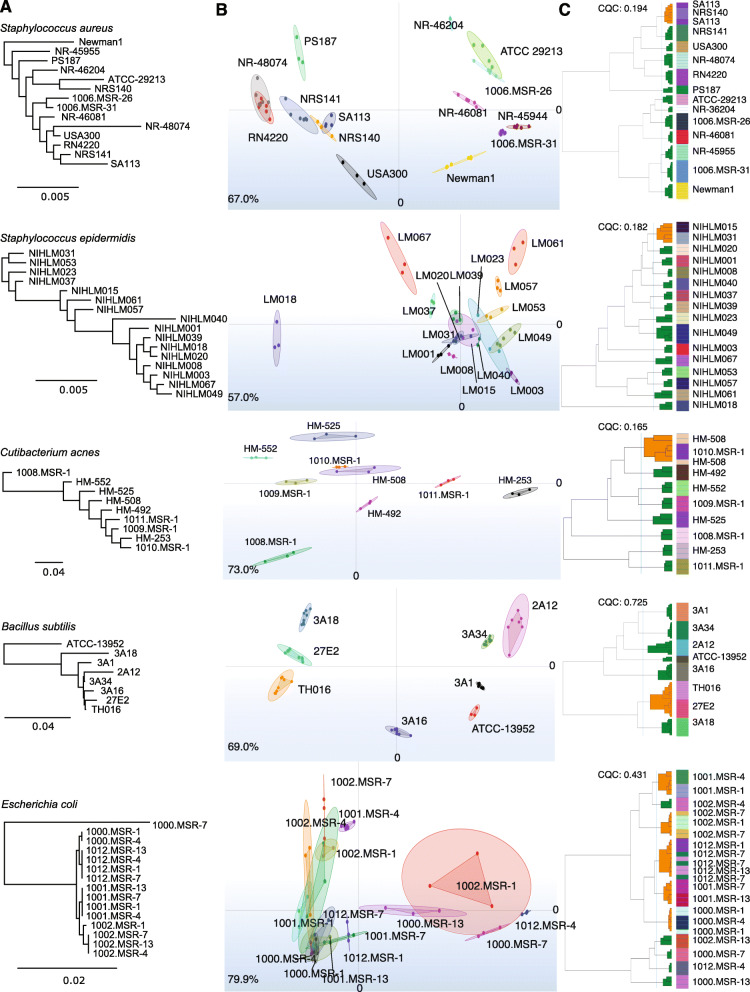
Biotyper IR differentiates genetically distinct strains. **A)** Phylogenetic trees of strain genomes tested in the Biotyper IR analysis based on alignment of bacterial marker genes. Genetic distance is shown in a dendrogram; genomes used (generated in this study or obtained from public repositories) are in Table S1. **B)** Principal component analysis (PCA) plot showing clustering of strains for each species, with each color representing a unique isolate and each dot within that color representing the isolate’s replicate spectral measurements. Links to the dots showing the variance of the technical replicates; output from IR Biotyper interface. ##% in lower left corner indicate the sum of variance explained by the first two principal components. **C)** Dendrogram of isolates based on spectral measurements; output from IR Biotyper interface. Green and orange in dendrogram represent cluster purity as determined by the Bruker IR software, based on technical replicates of strain spectra: green (“GOOD”), orange (“BAD”). Cluster quality criterion (CQC) indicates how well replicate measurements of an isolate cluster with themselves as well as the purity or homogeneity of each cluster

We investigated IR’s ability to differentiate genetically diverse strains of *S. aureus, S. epidermidis, C. acnes* as important skin microbes, and *E. coli* and *B. subtilis* from the gut*.* We primarily investigated diverse isolates obtained from different individuals, with the exception of *E. coli* in which we investigated within-individual diversity (or clonality) by typing multiple isolates obtained from 4 individuals, each with at least 3 technical replicates (i.e., multiple ‘spots’ of the same colony). To identify general concordance between the phylogenetic distance (genomics, Fig. 3A) and biochemical distance (IR) between isolates for each species, we performed an exploratory analysis comparing dendrograms (Fig. 3C) and principal component analysis (Fig. 3B) generated from both datatypes.

Nearly universally, technical replicates were the most similar to each other, irrespective of species and supporting IR’s reproducibility. For each species, we give some examples of concordance and discordance of clusters formed by IR vs. genome sequencing (Fig. 3).

1. In most cases, *S. aureus* strains formed distinct clusters; in particular, *S. aureus* PS187, Newman1, and NR-45944 which were also identified as more distant phylogenetically. However, in cases where overlapping clusters were identified by IR (e.g., 1013.MSR-26 & ATCC 29213, SA113 & NRS140, and RN4220 & NR-48074), most were relatively genetically distinct. In some cases, genetic and IR clusters were recapitulated (NRS141 & SA113, NR-46204 & ATCC-29213). Surprisingly, 1006.MSR-31 and 1006.MSR-26 were both highly genetically similar (isolated from the same individual) but did not form overlapping clusters.
2. We observed two clades of *S. epidermidis* strains, consistent with our previous large-scale genome analysis [7]. However, these clades were not recapitulated by IR, with the most distinct IR clusters coming from more genetically related strains (NIHLM018, NIHLM067, NIHLM061). Even highly genetically similar strains (e.g., NIHLM018 and NIHLM020), formed distant and distinct clusters.
3. *C. acnes* strains, each isolated from different individuals, largely formed distinct clusters with the exception of 1008.MSR-1 and HM-508 which shared overlapping clusters, but were genetically distant. HM-253 and 1010.MSR-1 were genetically most similar but were more closely related to other strains by IR spectra.
4. The *B. subtilis* strains tested had relatively higher genetic distance than the previous species. TH016 and 27E2, which were genetically most similar, formed overlapping IR clusters, but 3A18, which was relatively more distant, was also a near neighbor. ATCC-13952 robustly formed a distinct cluster and was an outlier genetically. In the case of *B. subtilis,* we also tested biological replicates (independent colonies between two separate runs), which yielded highly concordant results with strains from both runs grouping into the same cluster(s) (Fig. S3).
5. The ability to differentiate *E. coli,* where we examined both within- and between-individual variation, differed from the other species tested in its relatively lower clarity in strain differentiation by IR. However, upon closer look, in most cases, strains from the same patient clustered together based on their spectral profile (e.g., 1001.MSR-4 & 1001.MSR-1, 1002.MSR-4 & 1002.MSR-1 & 1002.MSR-7, 1012.MSR-1 & 1012.MSR-7 & 1012.MSR-13, 1000.MSR-1 & 1000.MSR-4). Because *E. coli* within an individual likely derives from a single lineage (like *B. fragilis* in the gut [54]), this underscores a strength of the IR in its ability to discriminate clonal strains from other strains.

A surprising result was 1000.MSR-7, which had been identified as *E. coli* by MALDI-TOF, and which genetically was relatively distant from the other *E. coli* strains (taxonomic identification based on alignment of a set of core marker genes), including other strains from the same patient, but shared an IR spectral cluster with another patient’s strain (1002.MSR-13). Upon closer examination of the whole genome sequence, this strain, which had been identified as *E. coli* by MALDI-TOF, was classified as *Klebsiella pneumoniae*, which shares 99.01% ANI (average nucleotide identity) in the single copy marker genes used for classification. This particular discrepancy is mostly likely attributable to a limited ability of MALDI-TOF to differentiate these species. Anecdotally, we have observed similar results when testing different strains from different species on the IR (data not shown). This reinforces the need for strain isolation coupled with rapid and low-cost approaches that differentiate the strains by either genomic or phenotypic features.

## Discussion

Vis a vis our general cultivation pipelines, we note two general observations. First, we observed a significantly different recovery rate for many microbes. For example, staphylococci in the skin, *E. coli* and *Enterococcus* in the gut, and streptococci in the oral cavity are recovered with far greater frequency and repetition than more abundant but more fastidious microbes. Second, we found that most microbes could be recovered on multiple growth conditions, and that there were relatively few media that specifically allow recovery of a desired taxon. For example, selective staphylococcal media (e.g., SaSelect culture plates) is designed for colorimetric differentiation of staphylococci, but frequently recovers *Bacillus* and *Micrococcus.* Anecdotally, we have explored depletion of such microbes like staphylococci by antibiotics, lysostaphin, and crystal violet, which resulted in low-to-moderate depletion, and per the goals of the project at hand, are recommended for further exploration. Overall, we believe that our approach results in broad recovery of abundant bacteria and low-abundance, non-fastidious bacteria, and as such, this general philosophy may be must useful for recovering and rapidly differentiating patient-specific strains of these microbes. For deeper recovery of the microbial diversity in a sample, we thus recommend on a per-application basis, evaluation of targeted approaches for the recovery of desired microbes, either via depletion of abundant microbes, increased number of growth conditions, and potentially most importantly, increased numbers of colonies surveilled. In addition, some microbes are epibionts, requiring co-culture for growth e.g. [25],. In addition, an increasing number of innovative approaches, including engineered antibody capture [25], microfluidics devices that can be placed in the environment [24], 3-D organoids to better recapitulate growth environments, and high throughput content screenings, are being developed to facilitate increased recovery of desired microbes for follow-up experimentation.

For strain identification, our results suggest that IR’s ability to differentiate strains is first, likely species-specific. This means that different species can have notably different levels of strain-level genetic diversity, i.e., some bacteria have relatively closed vs. open pangenomes, e.g., *C. acnes* [55] and *S. epidermidis* [7]*,* respectively. That may differentially affect cell surface macromolecule diversity and hence the IR readout. Second, IR is likely most valuable in differentiating clonal strains from any other non-clonal strain, rather than making a general assessment of genetic divergence and phylogenetic placement. In this way, we found that the IR had particular value to our goals of selecting unique strains from dominant microbes. In cultivating dominant microbes, we typically observe ‘frequent fliers’, i.e., microbes cultivated repeatedly. However, from MALDI-TOF it is impossible to determine if these are clonal both within *and* between individuals. For example, *Bacteroides fragilis* has been deemed relatively clonal in the gut [54], and thus selecting just one representative isolate per individual might suffice, but for *S. epidermidis*, which has extensive within-individual strain diversity [7], several representative strains might be chosen. The IR provides sufficient speed and resolution to discriminate clonality, particularly if benchmarked to a reference strain.

It is important to also note limitations of the IR. For example, run-to-run reproducibility in differentiating the same set of strains was strong (Fig. S2), but it is difficult to directly compare between runs, particularly with a large strain set. This is because different sets of strains from different runs are analyzed within-run, rather than benchmarked to an external database. Thus, given the 96-spot format, ~ 30 strains can be typed simultaneously accounting for technical triplicates. Second, as we previously noted, real genetic distance is difficult to deconvolute without an extensive paired comparative genomics approach. Nevertheless, despite these limitations, we believe that this rapid strain differentiation would be useful for selecting a subset of isolates from a set of patient cultivars that minimizes likely phenotypic redundancy.

Finally, we comment on practicalities for deploying these approaches. In mid-2021 costs with intermediate personnel, we estimated the stool pipeline described herein to cost ~$450/sample, reflecting the relatively greater reagent costs and extensive person-hours (~ 10 person hours) with the numerous conditions and limited multiplexing (4 samples at a time). Oral and skin sites were significantly lower in both costs and labor at ~$125/sample (~ 2 person hours/site), though these numbers benefit from scalability as again, we seldom cultivated from a single skin or oral site. However, there is significant value in using MALDI-TOF and IR technologies, as a set of 96 species or 30 strains can be profiled in 30 or 60 min for $15 and $175, respectively, at 2021 reagent costs using a 2016 machine. In contrast, 16S rRNA sequence classification or whole genome sequencing approximates $10/sample and ~ $30–$50/genome (extensively multiplexed on Novaseq S4) for reagents alone, respectively, and is at its most rapid, 24h hour turnaround time for 16S sequencing and ~ a week for a fully analyzed genome sequence.

## Conclusions

Here, we have presented and characterized a facile workflow for cultivation of bacteria from skin, oral, and gut microbiota from genus/species to the strain level with a focus on throughput across many patient samples rather than comprehensive recovery of all the species within a sample. We believe these efforts add to an increasing body of approaches for translating genomics-driven discoveries to microbial mechanism, and highlights the value-add of strain-level analysis to better understand genetic and phenotypic diversity underlying host-microbiome interactions.

## Methods

### Sample acquisition

Forehead and toeweb swabs (skin) and inner cheek and dorsum of the tongue (oral) swabs, and stool samples were obtained from our internal repository of human samples (approved by the Jackson Laboratory Institutional Review Board). Altogether in this study, we used 25 different samples obtained from 12 individuals. For skin and oral microbiota, sites were swabbed rigorously using 2 PurFlock Ultra buccal swabs (Puritan™ PurFlock Ultra, #22–029-506) for each site for 30 s before one swab was submerged into a 1.5 mL Eppendorf tube containing 500 μl Reasoner’s 2A (R2A) broth culture media (Lab M, LAB203-A) and the other into a microfuge tube containing 350 μL Tissue and Cell Lysis buffer (Epicentre, MPY80200) and 100 μg 0.1 mm zirconia beads (BioSpec Products, 11,079,101) for whole-genome shotgun metagenomic sequencing. Stool was self-collected at home by volunteers using a BioCollector fecal collection kit (The BioCollective, Denver, CO) according to manufacturer instructions. The volunteers also added a portion of the stool sample to an OMNIgene•GUT tube (DNA Genotek, OMR-200) following manufacturer instructions for preservation for sequencing prior to sending the sample in a provided Styrofoam container with a cold pack. Upon receipt, stool and OMNIgene samples were immediately aliquoted and frozen at − 80 °C for storage. Stool samples were homogenized inside the kit sample transport bag manually by massage and compression until combined (5–10 s), then roughly 1 mL was aliquoted into microfuge tubes. Prior to aliquoting, OMNIgene stool samples were homogenized by vortexing (using the metal bead inside the OMNIgene tube), then divided into 2 microfuge tubes, one with 100 μL aliquot and one with 1 mL.

### Cultivation conditions (Table 1)

#### Cultivation approach

*Skin and oral*. The 1.5 mL Eppendorf tube containing R2A and swab was thoroughly vortexed, and then diluted 1:100 and 1:1000 in R2A, to increase the chance of recovering single colonies. 50 μL from each dilution was then spread on half of an agar plate for each cultivation condition using sterile glass beads (Fisher Scientific, 50–444-635) or a sterile spread tool (Thomas Scientific, 229,616).

*Stool.* Approximately 5 g of stool sample was thawed and added to a 50 mL conical tube containing either 15 mL PBS (for aerobic culture) or 15 mL deoxygenated PBS with 0.1% L-cysteine (Sigma-Aldrich, 168,149) (for anaerobic culture), then vortexed well for 5 min and left to settle for 15 min. For the anaerobic direct plating condition, serial PBS/L-cysteine dilutions of 1:10,000 and 1:100,000 of each sample were plated on gut microbiota medium (GMM) plates [3] and left to incubate at 37 °C degrees in the anaerobic chamber for 48–72 h until colony formation was observed. Blood culture-assisted cultivation was utilized to select for the growth of underrepresented and slow-growing species, the PBS/stool mixture was added to a variety of culture conditions and incubated for 3, 7, 14, and 28 days prior to being diluted in PBS/L-cysteine and plated on blood agar plates (Table 1 and Fig. 1). Plates were incubated in the atmosphere and temperature of the original culture for 24–72 h until colony formation was observed.

#### Media and supplemental items

For liquid and agar cultivation as detailed in Table 1, the following mediums were sourced from Fisher Scientific: Luria Broth (LB) agar (BP1425500), R2A agar (R454372), Trypitic Soy Broth (TSB) (DF0370-17-3), Brain Heart Infusion (BHI) (DF0037-17-8), Chocolate agar (B21169X), Buffered Charcoal Yeast Extract (BYCE) agar (B21808), Tryptic Soy Agar (TSA) with 5% sheep blood agar (B21261X). Brucella agar was sourced from Anaerobe Systems (AS-141), while MacConkey and Selective Strep agars were purchased from Hardy Diagnostics (GA35 and A70 respectively). The supplemental additions included sheep’s blood (Fisher Scientific, R54008), rumen fluid (Fisher Scientific, NC9821770), vancomycin (Sigma-Aldrich, V1139) and colistin (Sigma-Aldrich, C4461). Aerobic and anaerobic blood bottles were purchased from Fisher Scientific (23–032512 and 23–032513 respectively). The 5 μm filters were purchased from Fisher Scientific (SLSV025LS).

#### Environments

The anaerobic atmosphere consisted of 5% hydrogen, 5% carbon dioxide, 90% nitrogen (Airgas, Z03NI9022000008). Aerobic cultures were conducted in ambient atmosphere.

### Isolate identification

#### MALDI-TOF

We used matrix assisted laser desorption ionization-time of flight (MALDI-TOF, Bruker Daltonics, Germany) mass spectrometry to identify isolates. Ten to twelve single colonies from each cultivation condition were picked and replated onto a new blood agar plate (Fisher Scientific, B21261X), then grown for 24–48 h to generate sufficient material for identification and archiving. Using a sterile transfer device (Puritan, 25–28,107), bacteria were directly transferred to a MALDI target spot. We then used the ‘extended direct method’ for sample preparation, in which 70% formic acid (Sigma-Aldrich, 5,438,040,100) is used to solubilize the bacterial cell wall prior to addition of matrix (Bruker Matrix HCCA, 14932). One spot on the target was reserved for the bacterial test standard (Bruker, 8,255,343) for calibration. Mass spectrometry analysis was performed using Flex Control 3.4 software (Bruker Daltonics, Germany). Identification score values below 1.60 were considered failed. Colonies not recognized by MALDI-TOF were processed using the ‘protein extraction’ method, and failing that by 16S rRNA gene sequencing as detailed below.

#### Protein extraction

Bacteria failing identification by the extended direct method were then extracted. 10 mg of biological material (generous scoop of a 1 μL inoculation loop) was thoroughly suspended in 300 μL of High-Performance Liquid Chromatography water (Sigma-Aldrich, 900,682) then thoroughly mixed with 900 μL 100% ethanol in a 1.5 mL Eppendorf tube. The tube was then centrifuged at 13,000 rpm for 2 min and the supernatant decanted. The tube was centrifuged again, and remaining ethanol was removed with a small pipet or allowed to air dry at room temperature. 10 μL of 70% formic acid was thoroughly mixed to the pellet by pipetting, followed by 10 μL acetonitrile (Sigma-Aldrich, 900,667), then centrifuged for another 2 min. 1 μL of supernatant was used on each MALDI target spot, dried, and overlayed with 1 μL of matrix. Mass spectrometry analysis was performed as above.

#### 16S rRNA sequencing

Bacteria failing identification by mass spectrometry and protein extraction were identified by 16S rRNA gene sequencing. Alkaline lysis was used to generate microbial DNA for PCR using universal primers 8F and 1391R (Turner, 1999). Sanger sequencing (GeneWiz, Inc) results were analyzed using BLAST (blast.ncbi.nlm.nih.gov). An identity score of 99% or higher was the threshold used for accurate species identification.

For archiving, the recovered bacteria were grown in TSB supplemented with 0.1 mg/L Vitamin K (Sigma-Aldrich, 95,271) and 5 mg/L hemin (Sigma-Aldrich- H9039) 24–48 h in 96-well plates at 37 °C in the appropriate atmosphere, then stored in 20% glycerol (Fisher Scientific, BP229–1) at − 80 °C.

#### Whole genome sequencing

Rapid DNA extraction from *S. epidermidis* isolates was adapted from Köser et al. (2014) and were performed as in Zhou et al. (2020) [7]. Briefly, 1 mL of overnight culture was centrifuged at 20,000 x g for 1 min. The bacterial pellet was then resuspended in 100 μL of 1X TE-buffer and transferred to a 2 mL bead beating tube with 100-125 μL 0.5 mm diameter glass beads (BioSpec Products, NC0417355) for homogenization. An extra 100 μL of 1X TE-buffer was added to the tube, which was then vortexed for 30 s at 3000 rpms using a vortex adaptor (Mo Bio Laboratories). Tubes were then centrifuged at 13,000 x g for 5 min to pellet the majority of cellular debris. The majority of the supernatant, taking care to not disturb the pellet, was then transferred to a fresh tube for quantitation of DNA using a Qubit 2.0 Fluorometer (Thermo Fisher Scientific). Supernatant was then diluted to 160 pg/μl. To make Nextera XT libraries, we used the Illumina standardized protocol (Nextera XT DNA sample preparation kit, Illumina Inc., FC-131-1096), creating dual indexed paired-end libraries. We adapted and miniaturized this protocol by taking all reagents in 1/4th amount and using 200 pg of DNA for each reaction, to generate an average insert size of 400 bp. Tagmentation and PCR reactions were carried out according to manufacturer’s instructions, and the resultant libraries were sequenced with 2X150bp paired end reads on an Illumina HiSeq2500 targeting ~ 5 million paired-end reads per sample.

### Metagenomic sequencing

#### Skin and Oral

We used our established protocol for metagenomic extractions [7]. DNA from swabs stored in lysis buffer and glass beads was extracted using the GenElute Bacterial DNA Isolation kit (Sigma-Aldrich, NA2110-1KT) with the following modifications: 5 μL of Lysozyme (10 mg/mL, Sigma-Aldrich, L6876), 1 μL Lysostaphin (5000 U/mL, Sigma-Aldrich, L9043) and 1 μL Mutanolysin (5000 U/mL, Sigma-Aldrich, M9901) were added to each sample, allowed to digest at 37 °C for 30 min. Then, samples were homogenized by bead-beating in a TissueLyser II (QIAGEN) for 2 × 3 min at 30 Hz. 5 μL of proteinase K (20 mg/mL, Sigma-Aldrich) and 300 μL of Solution C was then added and samples incubated at 55 °C for 30 min. 300 μL of 100% ethanol was used to precipitate the samples. Each sample was centrifuged for 1 min at 15000 x g prior to loading onto the GenElute column. Negative (environmental) controls and positive (in-house mock community of 26 unique bacterial species) controls were extracted and sequenced with each extraction and library preparation batch to ensure sample integrity. Subsequent steps were executed according to manufacturer instructions.

#### Stool

Approximately 50 μL of thawed OMNIgene preserved stool sample was added to a microfuge tube containing 350 μL Tissue and Cell lysis buffer and 100 μg 0.1 mm zirconia beads. Metagenomic DNA was extracted using the QiaAmp 96 DNA QiaCube HT kit (Qiagen, 5331) with the following modifications: each sample was digested with 5 μL of Lysozyme (10 mg/mL, Sigma-Aldrich, L6876), 1 μL Lysostaphin (5000 U/mL, Sigma-Aldrich, L9043) and 1 μL oh Mutanolysin (5000 U/mL, Sigma-Aldrich, M9901) were added to each sample to digest at 37 °C for 30 min prior to the bead-beating in the in the TissueLyser II (Qiagen) for 2 × 3 min at 30 Hz. Each sample was centrifuged for 1 min at 15000 x g prior to loading 200 μl into an S-block (Qiagen, 19,585) Negative (environmental) controls and positive (in-house mock community of 26 unique species) controls were extracted and sequenced with each extraction and library preparation batch to ensure sample integrity.

Sequencing adapters and low-quality bases were removed from the metagenomic reads using scythe (v0.994) [56] and sickle (v1.33) [57], respectively, with default parameters, as we have previously performed [7]. Host reads were removed by mapping all sequencing reads to the hg19 human reference genome using Bowtie2 (v2.3.1) [58], with “very-sensitive” parameters. Non-human reads (i.e., microbial reads) were used to estimate the relative abundance profiles of the microbial species in the samples using MetaPhlAn2 [49] (database downloaded 3/2020).

To identify *Enterococcus* species in metagenomic samples, stool metagenomes were mapped directly to reference genomes of *E. durans* (strain 8 L1–82), *E. avium (*ATCC 14025), *E. faecalis* (strain 39EA1) and *E. faecium* (ATCC 8459 = NRRL B-2354) using bowtie2 (version 2.4.1, −very sensitive mode) [58] extracting mapped reads using samtools (version 1.10 [59]) and then blasting to the respective species using standard parameters. Percent sequence identity was taken from the BLAST [60] results. The same pipeline was successfully applied for *C. acnes* and the skin samples as positive control.

### Strain typing with Biotyper IR

The Bruker IR Biotyper Fourier Transform Infrared (FT-IR) Spectroscopy system (Bruker Daltonics, Germany) was used to evaluate strain differences between isolates of a given species. The IR Biotyper analyzes the spectra of peaks corresponding to cell surface glycoproteins and uses hierarchical clustering to establish relationships between strains. Strains were grown from single colonies to a state of confluent growth on tryptic soy agar (TSA) plates. An overloaded 1 μL inoculating loop of cell material was resuspended in 50 μL of 70% ethanol (Sigma-Aldrich) in a 1.5 mL Bruker suspension vial with inert metal cylinders, and vortexed to homogeneity. 50 μL of deionized water was added to the tube, and again vortexed. 15 μL of each isolate suspension was pipetted onto 4 spots of a silicon microtiter plate along with 2 spots each of Bruker Infrared Test Standards 1 and 2 (Bruker, 8,255,343). The plate was allowed to dry in a 37 °C incubator, then loaded into the IR Biotyper for analysis. Spectra were processed by the IR Biotyper software in the 1300-800 cm-1 wavelength, corresponding to the carbohydrate region. Each spectra was comprised of 521 different datapoints. For exploratory analysis to assess similarity of spectra, we used the default IR Biotyper software settings to generate principal components analysis (PCA) plots and dendograms via hierarchical clustering using Euclidean distance to generate distance matrices.

### Comparative genomics of microbial genomes

WGS reads from isolate genomes (see “*Whole genome sequencing”*) were quality-filtered, trimmed, and assembled as described previously [7]. Briefly, sequencing adapters and low quality bases were removed from the sequencing reads using scythe (v0.994) [56] and sickle (v1.33) [57], respectively, with default parameters. Filtered sequencing reads were then assembled using SPAdes (v3.7.1) [61], with default parameters. The resulting draft genomes, as well as publicly available genomes (Table S1) were analyzed using the classify workflow (with default parameters) of GTDB-Tk (v1.0.2, reference database version r89) [62]. Based on the bacterial marker gene alignment generated by GTDB-Tk, a phylogenetic tree was inferred using FastTree (v.2.1.11) [63] with default parameters and visualized using Figtree (v1.4.4) [64].

### Additional statistical analyses

The frequency of cultivation was computed by counting the number of isolates identified under a given condition. For example, the frequency of the genus *Staphylococcus* for skin cultivation was the number count of all *Staphylococcus* species in that skin site, irrespective of the growth condition. Spearman correlations were computed in R with the base cor.test function. The rho value and the approximate p - values are shown in Table S5**,** example scatter plots in Fig. S2. Note here that because of modest sample size and sparse matrix, i.e., multiple zeros in our data (inherent to metagenomic analysis), R gives the warning ‘*Cannot compute exact p-value with ties*’.

#### Data deposition

Strains generated herein are available upon reasonable request. Genomes and metagenomic data are deposited in the Short Read Archive (SRA) under Bioproject PRJNA740337.

## Supplementary Information

**Additional file 1 Table S1.** Metagenomic read count statistics and genomes used in the study. **Table S2.** Relative abundances of bacteria, fungi, and viruses in metagenomic data. **Table S3.** Breakdown of genera recovered for each cultivation condition, by body site and timepoint. **Table S4.** Breakdown of species recovered for each cultivation condition, by body site and timepoint. **Table S5.** Spearman correlation between relative abundance in metagenomic data vs. frequency of isolation, genus- and species-level.

**Additional file 2 Fig. S1.** Relative abundance plots of oral, skin, and gut samples by genus, family, order, class, phylum. Each bar is an individual sample and the top 20 most abundant taxonomic features are plotted.

**Additional file 3 Fig. S2.** Example scatterplots underlying correlation analysis between frequency of species/genera cultivated and relative abundance by metagenomic analysis. Correlation of *Staphylococcus* at **A)** genus- and **B)** species-level.

**Additional file 4 Fig. S3.** Run-run reproducibility of Biotyper IR. For two individual runs of *B. subtilis* strains, shown are: **A**) PCA plot showing clustering of strains for each species, with each color representing a unique isolate and each dot within that color representing the isolate’s replicate spectral measurements. Links to the dots showing the variance of the technical replicates; output from IR Biotyper interface. **B)** dendrogram of isolates based on spectral measurements; output from IR Biotyper interface. Green and orange in dendrogram represent cluster purity as determined by the Bruker IR software, based on technical replicates of strain spectra: green (“GOOD”). Cluster quality criterion (CQC) indicates how well replicate measurements of an isolate cluster with themselves as well as the purity or homogeneity of each cluster.

## Data Availability

Strains derived are available upon reasonable request, and microbial genomes and metagenomic data is available on the Short Read Archive (SRA) under Bioproject PRJNA740337.

## Abbreviations

BCYE: Buffered charcoal yeast extract
BHI: Brain heart infusion
Fh: Forehead
rRNA: 16S ribosomal RNA
*C.*: *Cutibacterium*
CQC: Cluster quality criterion
*E.*: *Escherichia*
FT: Fourier Transform
IR: Infrared
GMM: Gut microbiota medium
IRB: Institutional Review Board
MALDI-TOF: Matrix assisted laser desorption ionization-time of flight
MLST: Multi-locus sequence typing
PCA: Principal component analysis
*S.*: *Staphylococcus*
sp.: Species
TD: Tongue dorsum
TSB: Tryptic soy broth
TSA: Tryptic soy agar
R2A: Reasoner’s 2A
