# Supplementary figures and images for: Cultivation of common bacterial species and strains from human skin, oral, and gut microbiota
Source: BMC Microbiol. 2021 Oct 14;21:278. doi: 10.1186/s12866-021-02314-y (PMC8515726; doi:10.1186/s12866-021-02314-y)

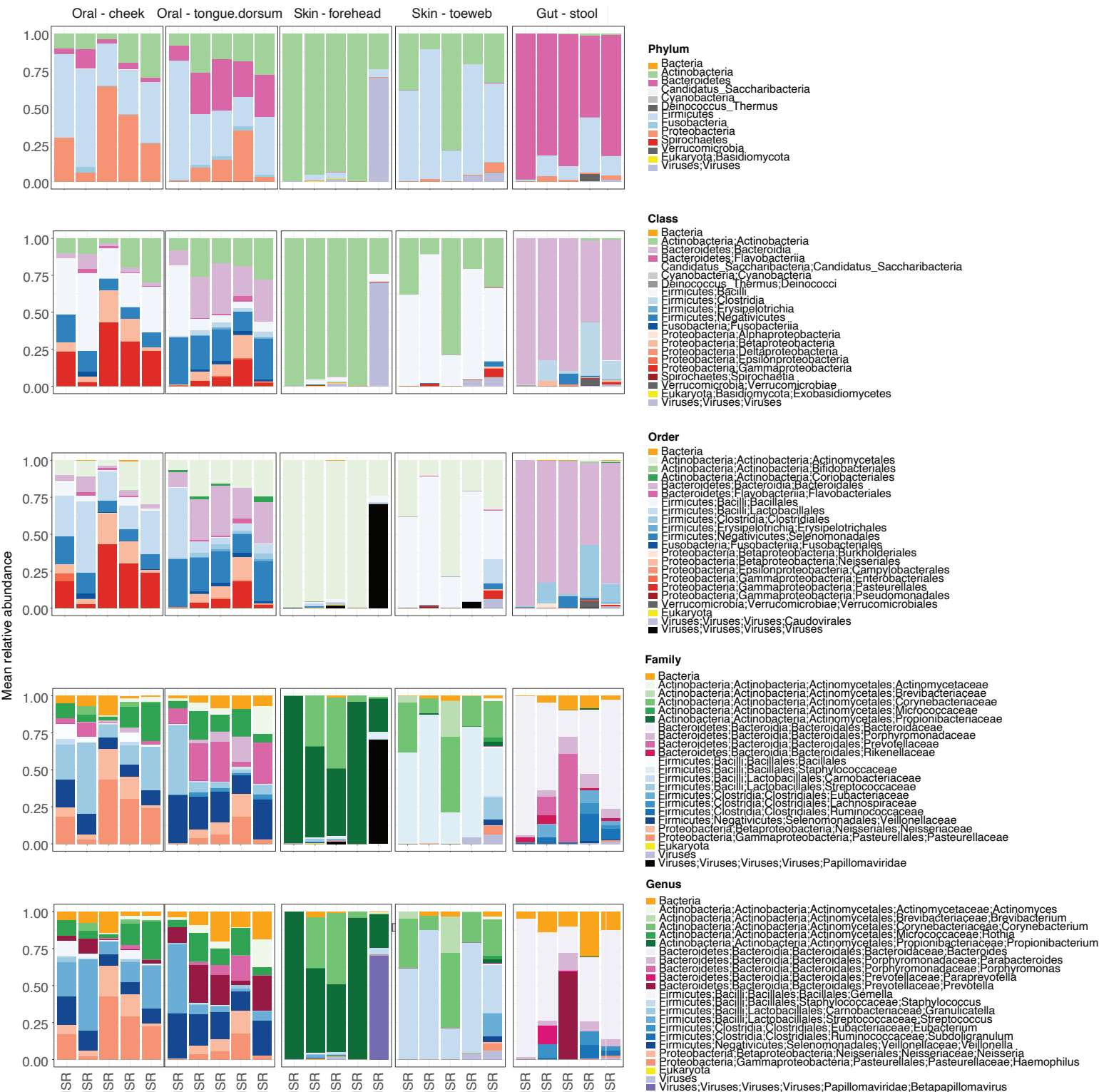

Supplement: Supplementary file 2 — Additional file 2 Fig. S1. Relative abundance plots of oral, skin, and gut samples by genus, family, order, class, phylum. Each bar is an individual sample and the top 20 most abundant taxonomic features are plotted. [file 12866_2021_2314_MOESM2_ESM.pdf]

**A**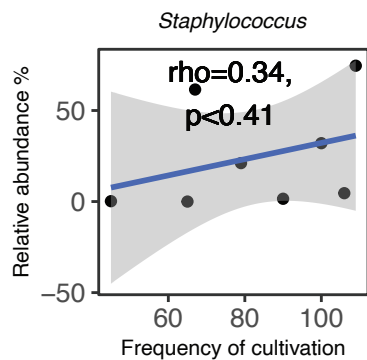**B**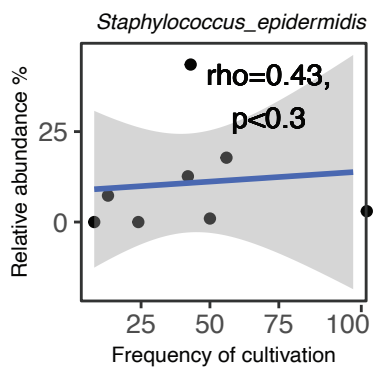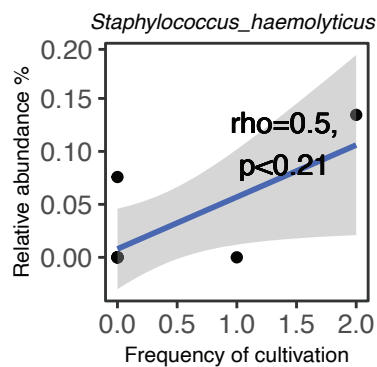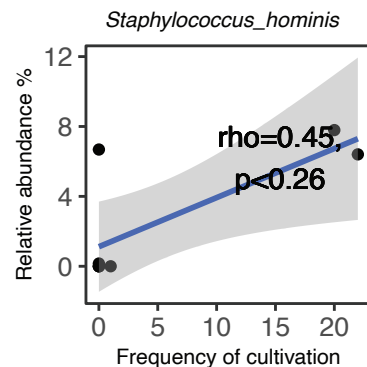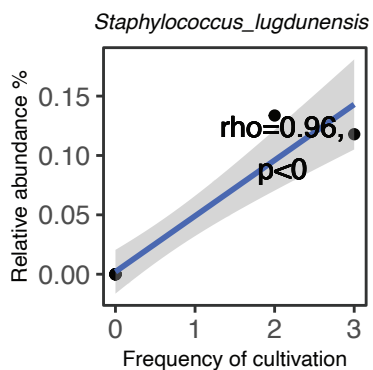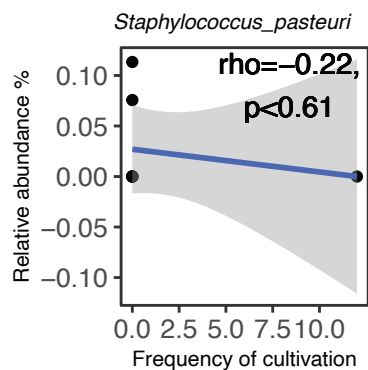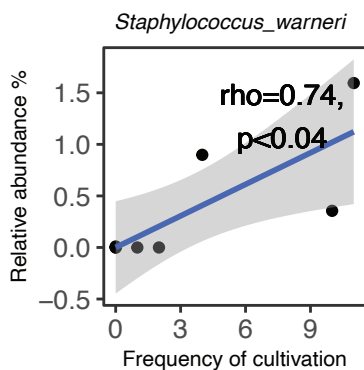

Supplement: Supplementary file 3 — Additional file 3 Fig. S2. Example scatterplots underlying correlation analysis between frequency of species/genera cultivated and relative abundance by metagenomic analysis. Correlation of Staphylococcus at A) genus- and B) species-level. [file 12866_2021_2314_MOESM3_ESM.pdf]

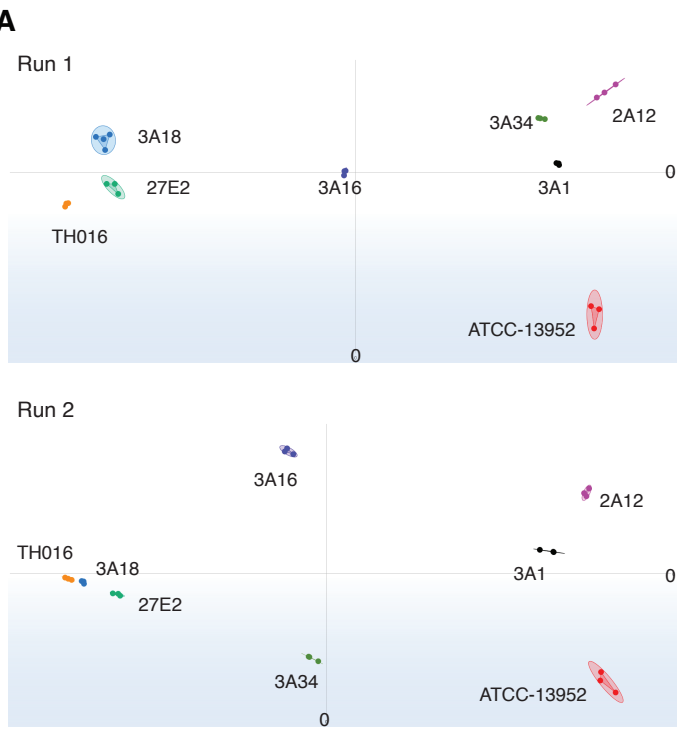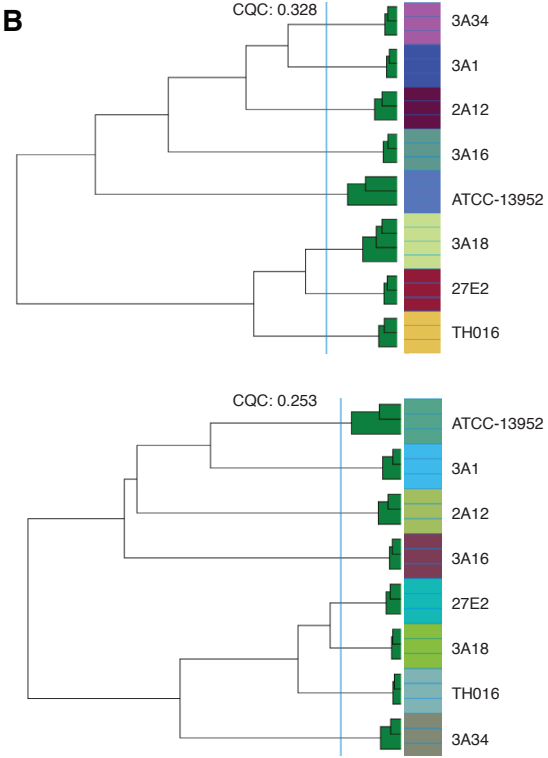

Supplement: Supplementary file 4 — Additional file 4 Fig. S3. Run-run reproducibility of Biotyper IR. For two individual runs of B. subtilis strains, shown are: A) PCA plot showing clustering of strains for each species, with each color representing a unique isolate and each dot within that color representing the isolate’s replicate spectral measurements. Links to the dots showing the variance of the technical replicates; output from IR Biotyper interface. B) dendrogram of isolates based on spectral measurements; output from IR Biotyper interface. Green and orange in dendrogram represent cluster purity as determined by the Bruker IR software, based on technical replicates of strain spectra: green (“GOOD”). Cluster quality criterion (CQC) indicates how well replicate measurements of an isolate cluster with themselves as well as the purity or homogeneity of each cluster. [file 12866_2021_2314_MOESM4_ESM.pdf]
